# The Human Scalp

**Published:** 1871-12

**Authors:** 


### THE HUMAN SCALP

Is often diseased, and intolerable headaches result from wearing the ordinary hat, which excludes the air altogether, aided by the custom of many, of keeping the hair plastered close down upon the scalp with the various forms of hair-oils and pomades, which occasion

### BALDNESS

in multitudes. It is of the utmost importance to the health of the hair, that the air should be allowed to have free access to every hair and to every root of it. The Indians wear no hats, and a bald headed Indian is seldom, if ever seen. Why has not

### THE GREAT HATTER, KNOX, been more on the alert, and devised a self-ventilating hat, such as J. E. Johnson & Co., of Philadelphia, have been using for some time, under their own patent. The little air holes in the centre of the top of some hats are but the most trifling expedient for ventilation; the hat of Johnson & Co. is the best ventilated, the lightest, and the most tasty of any yet made.

